# Self-reported selected zoonotic diseases among animal handlers in Urban Ahmedabad, India

**DOI:** 10.14202/vetworld.2019.176-182

**Published:** 2019-01-31

**Authors:** Krupali Patel, Deepak Saxena

**Affiliations:** 1 Center for Development Research (ZEF), University of Bonn, Germany; 2 Department of Epidemiology, Indian Institute of Public Health Gandhinagar, Gujarat, India

**Keywords:** animal handlers, knowledge and practices, self-reported zoonotic diseases

## Abstract

**Background and Aim:**

Out of all global microbial pathogens, 61% are zoonoses. Zoonotic diseases (Z/D/S) are responsible for a large burden on the public health, livestock economies, and wildlife of India. Data on burden and knowledge about Z/D/S among animal handlers are limited for urban and peri-urban areas of India. The present study aimed to estimate the prevalence of self-reported selected Z/D/S and knowledge about those diseases among animal handlers in the urban area of Ahmedabad city, India.

**Materials and Methods:**

This cross-sectional study was conducted among 170 animal handlers from three zones of Ahmedabad city, India, from February to May 2017. Data were collected on sociodemographic, different exposure, knowledge, practices about animal handling, and self-reported Z/D/S condition.

**Results:**

Majority of study participants were females. Participants had numbers of animals, and it ranged from 1 to 70. However, the majority of them were cattle. Average experience and hours/day spent for handling animal were reported 22±15 years and 5±2 h, respectively. From all participants, about one-third perceived that handling animal could be a cause of disease. Average knowledge on the mode of transmission of Z/D/S was found 4.1%. Most common high risk and preventive practices found consumption of raw milk (72%) and handwashing (83%). The proportion of self-reported Z/D/S in the past 5 years was found to be 23% among respondents and 17% among family members. However, the proportion of existing self-reported Z/D/S or symptomatic Z/D/S was 17% among respondents and 18% among family members. Most common self-reported Z/D/S were vector-borne, animal bite, and respiratory disorders.

**Conclusion:**

The knowledge and prevalence of Z/D/S were found low as compared to other studies from India. Further awareness and screening of animal handlers can be useful to increase the reporting and prevention and control of Z/D/S among them.

## Introduction

Any disease or infection that is naturally transmissible from vertebrate animals to humans and vice versa is classified as a zoonoses [1]. Globally out of all microbial pathogens, 61% are zoonotic with 13% species regarded as emerging or reemerging. Zoonotic diseases (Z/D/S) have great public health importance, as almost 75% of newly emerging infectious diseases are zoonoses [2].

Report from the International Livestock Research Institute in 2014 estimated that in developing the world around 1,000,000,000 livestock keepers affected by zoonoses [3]. Z/D/S are also responsible for a large burden on public health, livestock economies, and wildlife of India [4]. In India, 68% of the workforce relies on farming who are in close contact with domestic animals and poultry thereby having frequent exposure to sick or infected animals [5]. Moreover, Z/D/S are not limited to the only rural area. Rapid urbanization has led to megacities became the main container of incubators for new epidemics and Z/D/S, which can spread in a more hasty way and become worldwide threats. There are several reasons contributing to the occurrence of Z/D/S in urban areas, but rural migration is most important, they are more responsible for bringing their domestic animals to urban settings mainly in slums which encouraging an urban transmission as well as public health problems [6]. Several studies have shown the prevalence of innumerable known and important Z/D/S such as leptospirosis, rabies, and avian influenza. However, few high-risk cohorts within urban area required to explore for the insight of the current situation [7].

The burden and knowledge regarding Z/D/S among animal handlers not documented adequately in Indian context, especially in an urban agglomeration. Hence, the study aimed to estimate the prevalence of self-reported selected Z/D/S and knowledge about those diseases among animal handlers in the urban area of Ahmedabad city, India.

## Materials and Methods

### Ethical approval and Informed consent

The ethical permission sought from an ethical review board of Indian Institute of Public Health, Gandhinagar-Institutional Ethics Committee. Written informed consent was obtained from all participants of the study.

### Study design

A cross-sectional study was conducted among animal handlers of Ahmedabad city, Gujarat, India from February to May 2017.

### Study setting

This study was conducted in Ahmedabad city, the biggest city of Gujarat state, India. The total population of the Ahmedabad Urban Agglomeration (which also includes the region governed by Ahmedabad Urban Development Authority [AUDA]) is 7.2 million people [8]. Administratively city is divided into 6 zones and 64 wards. According to the census for the ninth plan, there are 30,737 rural families living in Ahmedabad. Out of those, 5.41% (1663 families) live below the poverty line [9]. A total of 439,843 people live in slums in the city [10].

### Sample size

The estimated sample size was calculated with 10% proportionate rate at 95% confidence interval (CI) and 1 design effect using OpenEpi sample size calculator which came 137. After adding 20% attrition rate, the final sample size was decided 170.

### Study sample selection

The convenient sampling method was used to recruit 170 animal handlers based on operational feasibility. All persons who are engaged in handling animals (such as cattle, buffalos, cows, goat, dog, hen, and sheep) interviewed from three different zones of Ahmedabad city (South, East, and New west zone). The zones were selected based on the animal population. According to verbal information of Cattle Nuisance Control Department (CNCD), New west zone has the highest animal population in the city followed by east and south zones.

### Data collection

Data collected using the pre-tested structured questionnaire in vernacular language. The salient aspects of the questionnaire were sociodemographic data, occupational history, history of animal contact including past also, previous exposure, medical history, and selected Z/D/S include rabies/animal bite, bovine tuberculosis (TB), Q-fever, brucellosis or any other self-reported Z/D/S or in the form of symptoms. Further, knowledge, attitude, and practices about management of exposure to Z/D/S were also asked.

### Statistical analysis

Data were entered using in MS Excel 2013 with a data entry template. The independent variables such as sociodemographic data, occupational history, animal history, previous exposure, and past-present medical history were reported inform of proportion. To understand the difference in characteristics of these independent variables among presence/absence of self-reported selected Z/D/S (includes rabies/animal bite, bovine TB, brucellosis, Q-fever, or any other self-reported selected Z/D/S), specific statistical test, i.e., Chi-square or Fisher exact test was used on the sample distribution. The significance level for the bivariate is p<0.05. Further odds ratio (OR) was performed to check the relationship between different exposures and self-reported existing Z/D/S or symptoms. This will help to identify the high-risk strata of this cohort. Data were analyzed using SPSS v.18.

## Results

### Sociodemographics

Out of all 170 participants, 57% were females and rest 43% was male. The majority (42.6%) of the respondents belonged to 26-40 years of age group with a mean age of 42±15 years. There were 44% of respondents illiterate, out of literate 50% studied up to primary or more. Almost 86% of respondents were married, and 89% of respondents were Hindu. Around one-third of respondents were below the poverty line. Majority of the population stay in their own house, and 53% resided in “pucca” house. Migration status, 17% of the population did not belong to Ahmedabad city migrated from a different rural area within state or other parts of India.

### Distribution of study population as per occupational history of animal handling

**Figure-1:**
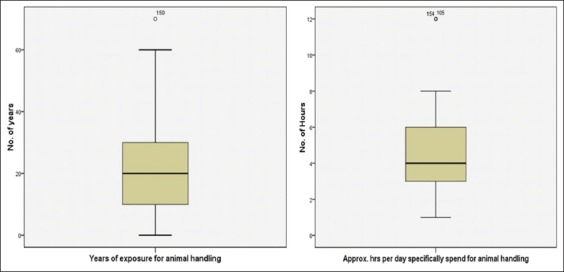
Exposures intensity.

Average experience of animal handling found 22±15 years and the median age of experience was found 20 years (Figure-1). The median value for numbers of animals was 5 (interquartile range=3-8) and ranged from 1 to 70 animals. Different types of breeds were reported including Buffalo 64%, Cow 38%, Goat 20%, Dog 5%, and Sheep and Bulls 4%. Respondents with hen and pigeons were found, respectively, 11% and 2%. Out of total respondents, 63.5% reared only one type of animal, but 37% had more than one type of animals. Respondents with different combinations of cattle and cattle with poultry animal were found, respectively, 76% and 24%. Residential location of the animal was mainly reported adjacent to compound (65%) in the form of open space. Only 8% of respondents reported separate shelter with a roof for the animal. Very few respondents kept their animal inside the house (4%), and all of them were poultry animal handlers. On the other side average, approximate hours spent for animals were found 5±2 h/day.]

### Knowledge and practices regarding Z/D/S

The study found that the knowledge toward zoonoses was poor; only 33% of respondents reported that handling animal could be a possible cause for acquiring the disease. Out of these, 30% had reported correct knowledge about zoonoses disease. Majority of them responded that rabies (11%), vector-borne disease (7%), bird flu (6.5%), Swine flu (2.4%), respiratory disease (1.2%), and skin disease (1.2%) could occur due to handling animals.

On inquiring about their knowledge on the various modes of Z/D/S transmission, it was found to be very poor as only an average of 4.3% knew about the modes of ZD transmission. Out of them, 7% and 0.6% and 6.5% and 7.6% reported that the disease could be transmitted through ingestion of raw milk and raw meat and through air and feed, respectively. Knowledge of transmission by contact with infected animal, contaminated excreta, contaminated water, and through animal bite was reported subsequently 5.3%, 2.4%, 0.6%, and 4.1, respectively.

Further inquiring about awareness regarding prevention from Z/D/S, 35.5% were aware about one or more than one methods. The most common method reported was as “Handwashing.”

### High-risk practices

**Figure-2:**
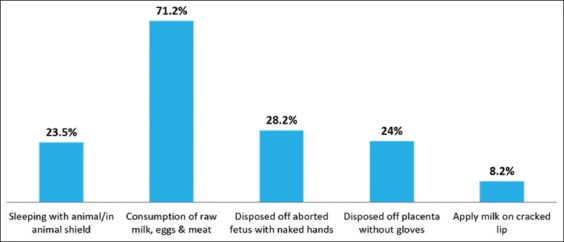
High-risk practices.

Few high-risk practices documented such as the practice of consumption of raw milk was highest (71%) when compared to consumption of raw meat and eggs found very less. The practice of applying raw milk on cracked lips was 8.2%. The practice of direct contact with animal products (without gloves/any barrier), placenta and aborted fetus were found 28.2% and 24% respectively. The practice of sleeping with animals/in-animal shield was reported by 23.5% as shown in Figure-2.

### Positive preventive practices

**Figure-3:**
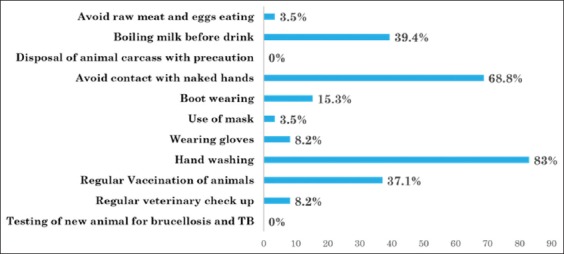
Preventive practices.

The practice of performing handwashing after finishing daily routine work of animal was reported to be the highest (83%) as shown in Figure-3. Although the majority of respondents (68.8%) reported practice of avoiding direct contact to placenta, practice of wearing gloves was reported only by 8.2%. The practice of using other barriers such as wearing boot and mask reported by 15.3% and 3.5%, respectively. Among them mainly male participants reported practice of wearing boot. On inquiring about the practice of regular vaccination, 37% reported practice of regular animal vaccination of animals but only 8.2% respondents reported that performed regular veterinary checkup of animals.

### Self-reported Z/D/S among animal handlers

Present study documented self-reported Z/D/S burden based on animal handlers’ perception.

#### Self-reported Z/D/S disease burden in the past 5 years (Table-1)

**Table-1:** Self-reported Z/D/S Burden of the past 5 years possibly linked to animal handling.

Variables	n=170 (%)
Self-reported Z/D/S burden at HH level	
With disease	55 (32.35)
Without disease	115 (67.65)
History of Self-reported Z/D/S among respondents	
Yes	39 (23)
No	131 (77)
Self-reported Z/D/S among respondents	
Chikungunya	27 (15.9)
Animal bite/Rabies	12 (7.06)
TB	1(0.6)
History of Self-reported Z/D/S among family members	
Yes	29 (17.1)
No	150 (88.2)
Self-reported Z/D/S amongst family members	
Chikungunya	22 (13)
Animal bite/Rabies	4 (2.35)
Swine flu	1 (0.6)
TB	4 (2.35)

Z/D/S=Zoonotic diseases, HH=Household, TB=Tuberculosis

Overall self-reported Z/D/S burden among household (HH) was found 32.35%. Further 23% respondents and 17% family members of respondents (irrespective of respondent disease condition) had a history of self-reported Z/D/S, which could be possibly linked to their occupation of animal handling. Proportions of self-reported Z/D/S among respondents were, respectively, vector-borne disease Chikungunya (15.9%), animal bite (7.06%), and respiratory disease (TB 0.6%). However, among family members were, respectively, vector-borne diseases Chikungunya (13%), animal bite (2.35%), and respiratory diseases such as TB (2.35%) and Swine flu (0.6%).

**Figure-4:**
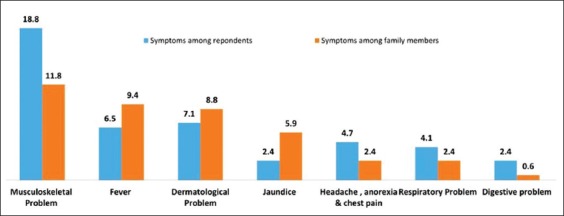
Symptomatic Z/D/S burden.

The second indicator of self-reported Z/D/S burden in the past 5 year was documented based on different types of Z/D/S symptomatic illness that could be possibly due to handling animals (Figure-4). There were 18.8% respondents reported that they were suffered from the musculoskeletal problem (such as joint pain and body pain). Further 7.1% of respondents had reported different dermatological problems among more common were itching and rashes. The respiratory problem (such as coughing, sore throat, and breathlessness) was reported 4.1%. Although fever is more common symptoms of any disease, only 6.5% respondents reported that they had a history of fever due to handling animals. Other symptoms reported were as a digestive problem (2.4%) and jaundice (2.4%).

Overall, low reporting is possibly due to a lack of awareness about zoonoses. Proportions of jaundice (5.9%), dermatological problems (8.8%), and fever (9.4%) were found relatively little more among family members.

#### Self-reported Z/D/S disease burden at the time of this study (existing Z/D/S burden)

It was observed that overall HH with existing disease condition was found at 31%. However, it was documented among respondents (17%) and family members (18.24%). Musculoskeletal problems (8.2%), dermatological problems (4.1%), respiratory problems (3.5%), fever and headache (1.8%), digestive problems (1.2%), and vector-borne disease (0.6%) were reported among respondents. However, burden of same among family members was found more which was reported musculoskeletal problems (7.1%), dermatological problems (6.5%), respiratory problems (4.1%), fever and headache (3.5%), digestive problems (6.5%), vector-borne disease (2.4%), and jaundice (1.2%).

### Relationship between different exposure and self-reported existing Z/D/S among respondents (Table-2)

**Table-2:** Relationship between different exposure and existing self-reported Z/D/S condition among respondents.

Variable	Category	Odds ratio (CI)
Gender	Female versus Male	0.771 (0.346-1.718)
Age	>40 years versus ≤40 years	2.971 (1.286-6.862)*
Marital status	Ever married versus single	1.228 (1.140-1.324)*
Education	Illiterate versus Literate	1.711 (0.765-3.825)
Total family members	>5 versus ≤5 family members	0.947 (0.426-2.106)
BPL card	Yes versus No	0.813 (0.334-1.975)
Immigrants in Ahmedabad	Yes versus No	0.742 (0.237-2.323)
Own source of water	No versus Yes	2.462 (0.785-7.724)
Access to toilet	No versus Yes	2.703 (1.141-6.403)*
Diet	Non-vegetarian versus Vegetarian	1.608 (0.644-4.020)
Type of animal	More than one type versus one	1.808 (0.807-4.054)
Animal residence	Out of compound versus inside house/compound	0.840 (0.345-2.043)
Animal Vaccination	No versus Yes	0.596 (0.247-1.439)
Approximate hours spent per day	>4 versus ≤ 4 h	1.459 (0.654-3.258)
Years of animal handling	>20 versus ≤ 20 years	1.924 (0.859-4.310)
Total numbers of animals	<=5 versus >5 Animals	0.994 (0.445-2.220)
Perception of animal handling could be a potential cause of disease	No versus Yes	2.2 (0.976-4.960)

Z/D/S=Zoonotic diseases, CI=Confidence interval,

* =p<0.05

Self-reported existing Z/D/S among respondents and its exposure pattern showed that some preventive practices have lesser odds of having disease. The odds of having disease among female found protective (OR=0.771; 95% CI: 0.346-1.718). Further, the odds of having disease due to handling animals was found significantly 2.9 times more among >40 years of age group (OR=2.971; 95% CI: 1.286-6.862). The odds of having disease due to handling animals was also found significantly 1.2 times more among ever-married group compared to single (OR=1.228; 95% CI: 1.140-1.324). Odds of having disease among illiterate found 1.7 times more compared to literate (OR=1.711; 95% CI: 0.765-3.825). Moving toward water and sanitation, the odds of having disease among no owned water source and no toilet access was found, respectively, 2.5 and 2.7 times more compared to those who had owned water source and toilet access. Relationship between sanitation and having disease due to handling animals was found significant (OR=2.703; 95% CI: 1.141-6.403). The odds of having disease due to handling animals among non-vegetarian was found 1.6 times more compared to vegetarian.

Animal handlers who have more than one type of animal have 1.8 times high odds of having disease. Persons who keep their animals out of compound were found protective from having disease due to handling animals (OR=0.840; 95% CI: 0.345-2.043). Further handlers whose animals got vaccines in the past year were also found protective from having disease due to handling animals (OR=0.596; 95% CI: 0.247-1.439). However, odds of having disease among handlers who spend >4 h with animals was found 1.5 times more. The odds of having disease among persons who are handling animals >20 years was also found 1.9 times more, but it is not significantly associated. Odds of having disease among animal handlers who do not have knowledge on handling animal could be a possible cause of disease was found 2.2 times more compared to who have knowledge on same (OR=2.2; 95% CI: 0.976-4.960).

## Discussion

The burden of ZD among animal handlers is not adequately reported for urban areas of Gujarat. In the present study, the overall percentage of self-reported Z/D/S among HH was found 32.35% (CI: 25-40%). The symptoms perceived by the community that can be attributed to ZD include musculoskeletal problems (8.2%), dermatological problems (4.1%), respiratory problems (3.5%), fever and headache (1.8%), and digestive problems (1.2%). Around 0.6% of respondents also opined that vector-borne diseases can be attributed to the presence of an animal in the vicinity. In a study conducted in Pondicherry 37.7% respiratory infection, 31.1% digestive disturbances, 15.5% dermatological problem, and 15.5% indiscrete diseases such as fever, body pain, and headache joint pain were reported as Z/D/S [11].

Knowledge about the risk of Z/D/S due to handling animal can contribute to the altered practices and occurrence of disease among animal handlers. In the present study, only 30% of the respondents had knowledge about the risk of ZD involved in their occupation which is very low. A recent study from India also reports limited knowledge of ZD among animal handlers (16.4%) [12]. The lack of knowledge could be the reason for the increased burden of diseases. Only 11% of respondents were aware about rabies being Z/D/S. About 7% consider vector-borne disease as Z/D/S. About 10% of the participants reported disease such as bird flu (6.5%), Swine flu (2.4%), respiratory disease (1.2%), and skin disease (1.2%) as also Z/D/S. The knowledge on rabies and bird flu is apparently low compared to other studies, for example, from Punjab shows, respectively, 84.8% and 92.8% [11,12]. This could be a missed link for health policymakers specifically in case of Ahmedabad as it has increased the burden of flue and rabies since past decade. Poor communication between the veterinarian and human health-care professionals is also responsible for low reporting as well as low awareness of zoonoses.

Present study documented high-risk practices that can possibly lead to occurrence of Z/D/S; such as consumption of raw milk (71%) disposing off aborted animal fetus with bare hands (28%), disposing off placenta without gloves (24%), and sleeping with/inside animal shelter (23.5%). Such practices are harmful and can increase the risk of getting different zoonoses and foodborne infections. Further, the finding was almost consistent with another study conducted in Punjab and Tanzania [13,14]. To prevent the many zoonoses transmission such as brucellosis, barriers method is most important. However, the practice of using gloves was found very low, consistent finding found in other studies but how many of animal handlers have a positive attitude toward barrier method is not adequately documented in the majority of the studies [14]. During the field visits, it was also observed that people use the stick as a barrier to avoid direct contact with the placenta.

Preventive practices are important which could reduce the risk of disease. In this study, the proportion of documented preventive practices were low as compared to other studies from India [14,15]. However, the prevalence of handwashing was found to be 83%, but the researcher of the present study observed that the majority of animal handlers wash their hands but do not wash every time when they touch their animal. It was also observed that all of them not washing their hands with the use of soap. They were using soap only after handling cow dung. Thus, some of the preventive practices are prevalent and followed by the community, but it needs to be improvised and promoted further.

## Conclusion

The frequency of people suffering from self-reported Z/D/S was found very less. In addition, animal handlers had very limited knowledge of Z/D/S and the ways to prevent them. Thus, proper screening of animal handlers for Z/D/S should be required to do by government or municipality. This will help to estimate the prevalence of Z/D/S among a high-risk group such as any animal handlers. Further results of this study also suggest that it is very crucial to generate the awareness on preventive practices of Z/D/S among communities to reduce the risk of Z/D/S and their transmission.

## Limitation of the study

Recall bias regarding S/S of Z/D/S could not be excluded. Further, results cannot be generalized since samples were largely from fix geographical area of AMC zones and convenient sampling methods were used. The estimated prevalence was based on S/S and self-reported conditions. Confirmatory diagnosis was not conducted due to lack of fund.

## Authors’ Contributions

KP: Conceptualized and designed the study, collected and analyzed data, drafted the manuscript. DS: Guided during conceptualization, designing and analysis of the study, reviewed the final draft. All authors read and approved the final manuscript.
